# Computational identification and characterization of noncoding RNA-encoded peptides: tools, databases, and *in silico* strategies

**DOI:** 10.1007/s00726-026-03531-3

**Published:** 2026-05-22

**Authors:** Nidhi Shenoy, Atharva Karkare, Varsha Gangadhar, Shashank Rao Padubidri, Sowndarya Rao, Leona Alison Dsouza, Manjunath B. Joshi, Rama Rao Damerla, Neha S. Gandhi, Sandeep Mallya

**Affiliations:** 1 Department of Bioinformatics, Manipal School of Life Sciences, Manipal Academy of Higher Education, Manipal, India; 2 Department of Ageing Research, Manipal School of Life Sciences, Manipal Academy of Higher Education, Manipal, India; 3 Department of Medical Genetics, Kasturba Medical College, Manipal Academy of Higher Education, Manipal, India; 4 Department of Biotechnology, Manipal Institute of Technology, Manipal Academy of Higher Education, Manipal, India

**Keywords:** ncRNA-encoded peptides, ncPEPs, Small open reading frames, sORFs, *In silico* peptide discovery, Coding potential prediction, Long noncoding RNAs

## Abstract

Once dismissed as transcriptional artifacts, noncoding RNAs (ncRNAs) have gained recognition in recent years for their ability to participate in gene regulation, as well as their ability to encode functional molecules referred to as ncRNA-encoded peptides (ncPEPs). The discovery of ncPEPs has opened new avenues in proteomics and genomics research, revealing biological mechanisms that were previously unexplored. This review presents an extensive overview of the computational tools, databases, and in silico strategies used to identify ncRNA-encoded peptides across all major ncRNA classes, including long noncoding RNAs (lncRNAs), circular RNAs (circRNAs), and primary microRNAs (pri-miRNAs). Furthermore, we outline publicly available databases that compile experimentally validated and computationally predicted ncPEPs across multiple species, enabling systematic annotation and cross-referencing of candidate peptides. By highlighting the current challenges and emerging methodologies, we emphasize how computational methods continue to advance our ability to uncover hidden functional peptides within the noncoding transcriptome. These developments provide a framework for validating ncPEPs and elucidating their biological significance across diverse systems.

## Introduction

The human genome contains only a small portion of protein-coding genes (approximately less than 2%), and the remaining portion is composed mainly of noncoding genes. Previously dismissed as junk DNA, many noncoding genes have been recognized as functional elements with crucial roles in cellular processes (Lander et al. 2001). It has been reported that a significant portion of this so-called junk DNA is actively transcribed into RNA. However, many of these transcribed RNAs exhibit minimal or no coding potential, suggesting that they may play noncoding roles (Yang et al. 2016). Studies have discovered small open reading frames (sORFs), which are small coding sequences in both canonical protein-coding transcripts and transcripts that were previously classified as noncoding. They usually encode peptides with fewer than 100 amino acids. Small proteins known as micropeptides are produced when sORFs are translated (Makarewich and Olson 2017). These peptides are frequently referred to as ncRNA-encoded peptides (ncPEPs) when they are particularly derived from transcripts categorized as noncoding RNAs (ncRNAs) (Chen et al. 2022). Consequently, ncPEPs are a subset of micropeptides derived from sORFs contained in transcripts annotated as ncRNAs. Despite their small size, ncPEPs are gradually becoming a new functional class of molecules that contribute to multiple cellular activities and are dysregulated in various disorders, contributing to pathogenesis (Dragomir et al. 2020).

The identification of ncPEPs requires a multifaceted approach that utilizes bioinformatics tools, databases, and experimental methodologies. Bioinformatics tools serve as indispensable aids in scanning genomic and transcriptomic datasets to identify potential sORFs within noncoding regions, considering features such as ORF length, nucleotide bias, internal ribosome entry site (IRES), and N6-methyladenosine (m6A) sites, among others. Several interactive and continually updated databases provide comprehensive repositories of experimentally validated and predicted ncPEPs, streamlining annotation efforts and guiding functional research. Experimental validation is essential for confirming translation, determining subcellular localization, and elucidating the roles of these peptides in cellular processes. Strategies such as ribosome profiling (Ribo-Seq) and global translation initiation sequencing (GTI-seq) offer invaluable insights into translation events by enabling the deep sequencing of ribosome-protected fragments and initiation sites, respectively (Ingolia et al. 2009, Lee et al. 2012). Proteogenomics, which integrates mass spectrometry (MS) with transcriptomic or genomic data, enables the identification and quantification of ncPEPs, thereby validating their existence and providing valuable insights into their expression levels and potential posttranslational modifications (Slavoff et al. 2013, Nesvizhskii 2014).

The recognition of ncPEPs embedded within ncRNAs holds immense potential in unraveling previously concealed functional elements, thereby expanding our comprehension of the intricate noncoding transcriptome landscape. These findings have potential for the discovery of novel regulatory mechanisms and cellular processes, thereby advancing our understanding of the underlying molecular intricacies governing biological systems. Hence, ncPEPs are promising targets for therapeutic interventions in diseases such as cancer. Several reviews have examined the biology and computational prediction of ncRNA-encoded peptides. Wu et al. (2020) provided an early synthesis of lncRNA- and circRNA-encoded peptides with a focus on their oncological roles, while Xing et al. (2021) and Pan et al. (2022) surveyed computational prediction methods and databases specifically for lncRNA-encoded micropeptides (Wu et al. 2020, Xing et al. 2021, Pan et al. 2022). However, these prior reviews address individual ncRNA subclasses and do not provide a unified, tool-by-tool evaluation framework spanning the full prediction pipeline. Unlike these prior works, the present review consolidates computational tools across all major ncRNA classes, including circRNAs, lncRNAs, and pri-miRNAs, into a single structured resource with explicit annotation of input requirements, species applicability, training dataset characteristics, and predictive performance metrics, thereby offering a practically oriented benchmarking framework for researchers seeking to select appropriate tools for their specific experimental and computational context. In this review, we explore tools, databases, and in silico methods for the identification and characterization of ncPEPs.

### Emergence of ncPEPs

Research at the beginning of the 21st century, following the completion of the Human Genome Project, revealed that much of the genome previously labeled junk DNA, accounting for approximately 98% of the total, is transcribed into ncRNAs. These transcripts, although they do not encode proteins, are recognized as key regulators of biological functions, such as the regulation of development and physiology and disease pathogenesis, and function as both tumor suppressors and oncogenes (Pavet et al. 2011). Hence, the abundance of ncRNAs is more strongly associated with human developmental and physiological complexity than protein-coding genes.

**Fig. 1:**
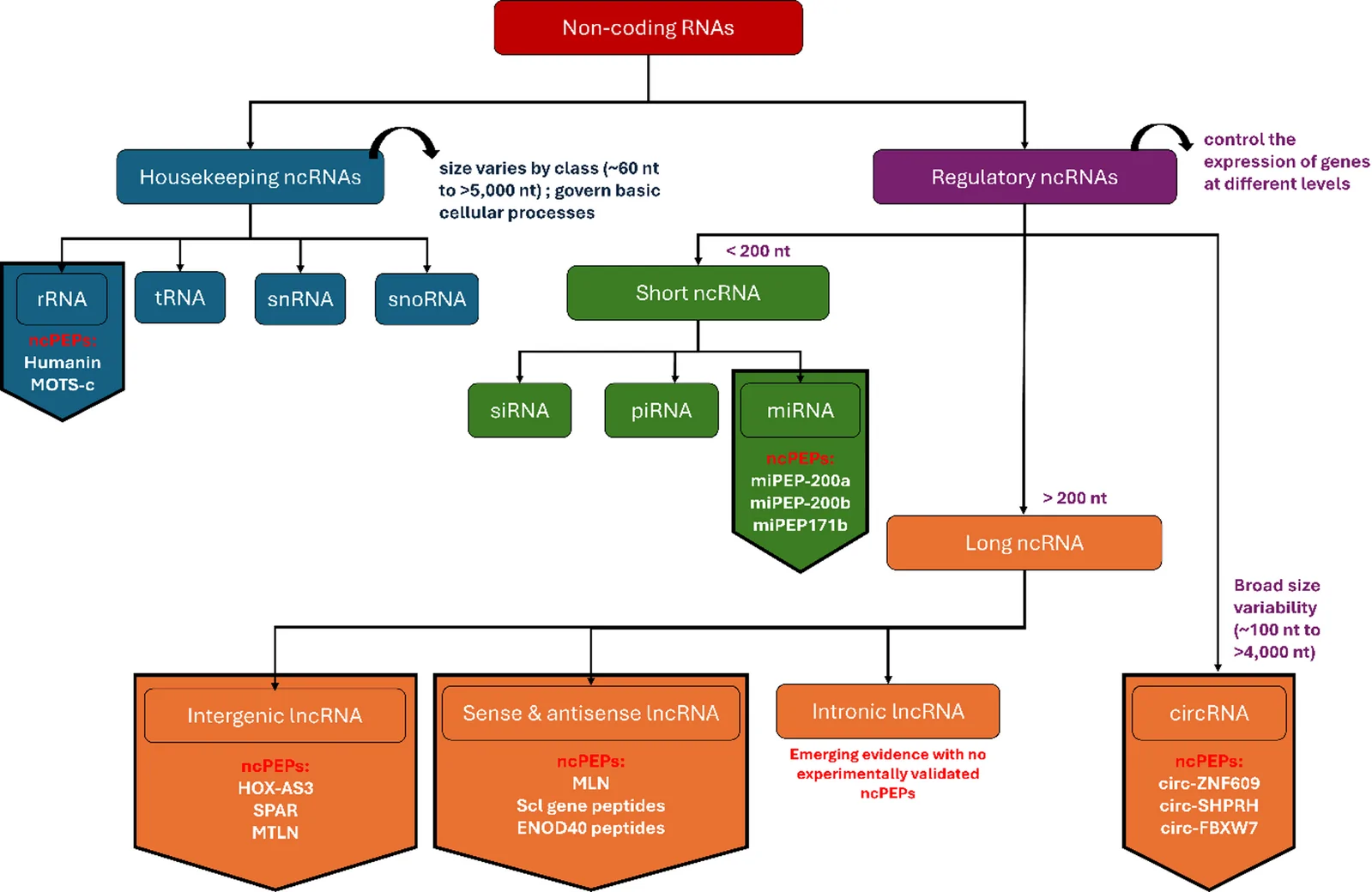
Classification of ncRNAs and their coding potential for ncPEPs. Specific classes of ncRNAs, including rRNAs, pri-miRNAs, intergenic lncRNAs, antisense lncRNAs, and circular RNAs (circRNAs), have been experimentally validated to encode ncPEPs, highlighted with representative examples. circRNAs are presented as a structurally distinct category defined by back-splicing, separate from lncRNA subclasses

ncRNAs are classified into two main categories: housekeeping ncRNAs, which include mainly rRNA, tRNA, snRNA, and snoRNA, and regulatory ncRNAs, which include miRNA, siRNA, piRNA, lncRNA, and circRNA (Zhang et al. 2019). Housekeeping ncRNAs are highly expressed and are primarily involved in fundamental cellular functions such as RNA splicing (snRNAs), RNA modification (snoRNAs), and protein synthesis (rRNAs and tRNAs). Their sizes vary considerably by class — snRNAs and snoRNAs are typically short (~ 60–400 nt), whereas rRNAs show broad size variability (~ 120 nt to > 4,500 nt). Regulatory ncRNAs modulate gene expression at the transcriptional, posttranscriptional, and epigenetic levels and are categorized into sncRNAs with lengths of less than 200 nucleotides and lncRNAs longer than 200 nucleotides (Zhang et al. 2019, Hashimoto et al. 2008). The key subclasses of sncRNAs include siRNAs, miRNAs and piRNAs, whereas lncRNAs primarily include circRNAs, lincRNAs, sense and antisense lncRNAs and intronic lncRNAs (Zhang et al. 2019, Chen et al. 2019). The detailed classification of ncRNAs, including their subtypes and coding potential for ncPEPs, is illustrated in Fig. 1.

Studies have identified numerous coding and noncoding transcripts with sORFs capable of encoding small peptides in eukaryotic genomes. These peptides are less than 100 amino acids in length and are widely distributed across species, cell lines and tissues (Dragomir et al. 2020, Li et al. 2017). Unlike conventional peptides derived from protein-coding genes, ncPEPs originate from the direct translation of sORFs within ncRNAs such as lncRNAs, circRNAs, and untranslated regions. These sORFs are often initiated with canonical AUG start codons but may lack conventional stop codons. Although conventional human proteins usually show at least panvertebrate and occasionally panmetazoan conservation, human sORFs may not be conserved beyond primates (Dragomir et al. 2020). Evidence suggests that ncPEPs play functional and regulatory roles in a multitude of cellular processes.

### Role of ncPEPs in biological processes

**Fig. 2:**
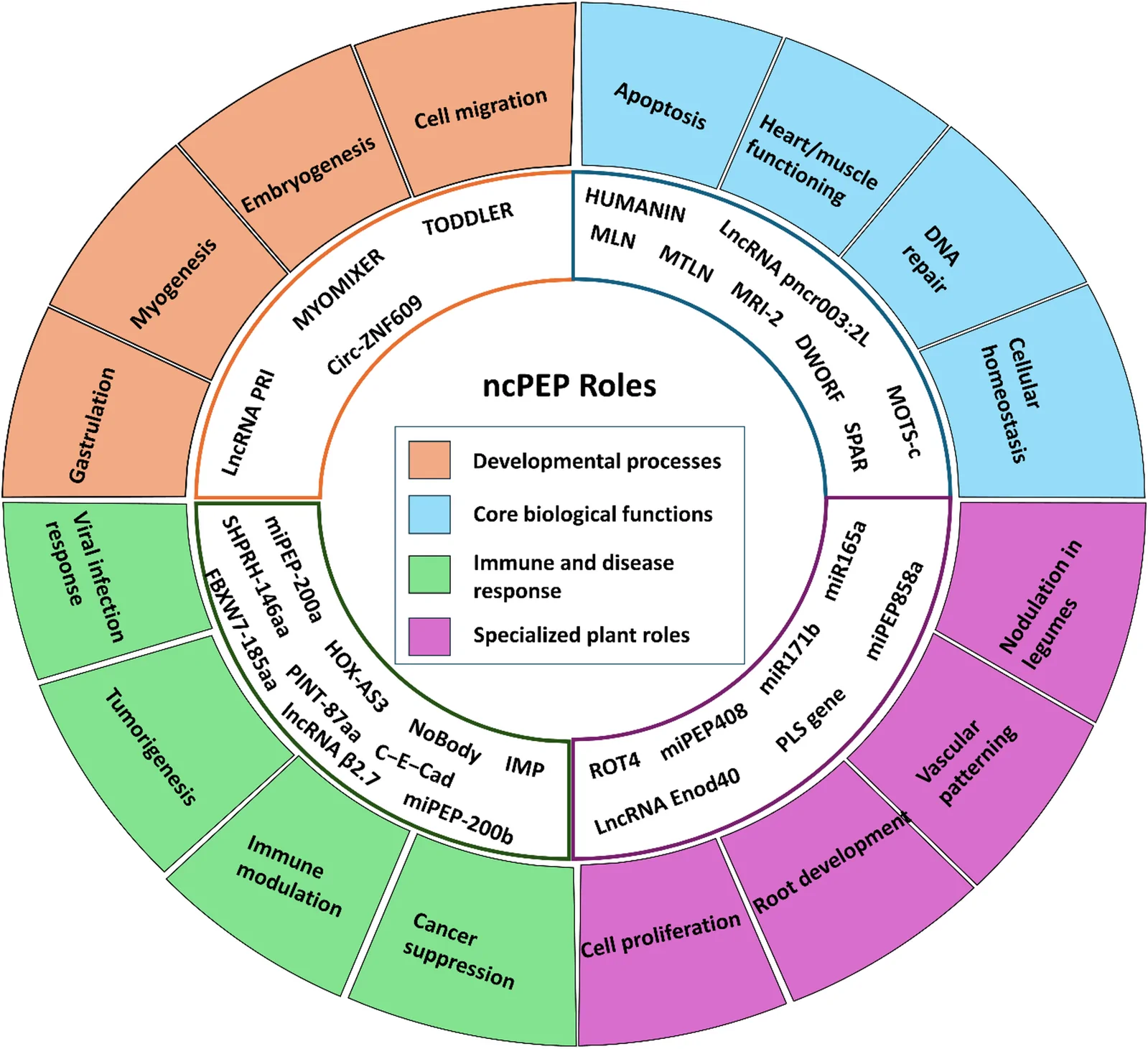
Overview of the functional roles of ncPEPs. The outer circle of the diagram depicts the key biological processes in different categories (differentiated by color), whereas the inner circle lists the specific ncPEPs and ncRNAs implicated in the respective category. The figure was created by the authors based on information compiled from published literature (Wu et al. 2020, Xing et al. 2021, Kong et al. 2020, Wang et al. 2023). Individual ncPEPs and their source ncRNAs are referenced in the corresponding sections of the text

Numerous studies have reported that ncPEPs encoded by lncRNAs, circRNAs, and miRNAs play pivotal roles in a wide array of biological processes. These peptides are recognized as critical regulators of development, core cellular functions, immune responses, disease progression, and plant biology, as depicted in Fig. 2.

### Developmental processes

The first animal-derived ncPEPs were identified in *Drosophila*, where the lncRNA *polished rice* (pri) or *tarsal-less* encoded small peptides ranging from approximately 11–32 AA, which are necessary for the development of flies during the embryonic stage (Hashimoto et al. 2008, Galindo et al. 2007, Kondo et al. 2007). In zebrafish, *Toddler*, a short, conserved peptide, functions as a motogen or signal that encourages cell migration by triggering G protein-coupled APJ signaling, promoting gastrulation (Pauli et al. 2014). *Myomixer*, a lncRNA-encoded 84 AA peptide, plays a crucial role in orchestrating myoblast fusion events during the developmental stages of skeletal muscle tissue (Bi et al. 2017). Similarly, *circ-ZNF609*, a circRNA that encodes a peptide involved in myogenesis in vertebrates, has been translated in a cap-independent manner (Legnini et al. 2017).

### Core biological functions

Several ncPEPs have been shown to regulate fundamental cellular activities. For example, the *sarcolamban* (scl) gene, previously annotated as lncRNA *pncr003:2 L*, encodes peptides essential for cardiac contraction in *Drosophila* (Magny et al. 2013). *Mitoregulin* (Mtln), a 56-AA peptide encoded by a putative lncRNA that is predominantly expressed in skeletal and cardiac muscle, plays a fundamental role in cellular energy production and homeostasis (Stein et al. 2018). Other ncPEPs, such as *Myoregulin* (MLN) and *DWORF*, play opposing roles, with the latter enhancing SERCA activity and muscle performance (Nelson et al. 2016).

*Humanin*, encoded within mitochondrial rRNA, regulates apoptosis by inhibiting Bax (Bcl2-associated x protein) translocation to mitochondria and exerting neuroprotective effects (Yeasmin et al. 2018, Matsuoka et al. 2006). The mitochondrial 12 S rRNA gene also encodes *MOTS-c*, a 16 AA peptide that influences metabolism, weight regulation, and insulin sensitivity (Lee et al. 2015). *SPAR*, a peptide encoded by the lncRNA *LINC00961*, influences lysosomal activity. It regulates amino acid signaling and prevents muscle regeneration by inhibiting mammalian target of rapamycin complex 1 (mTORC1) (Matsumoto et al. 2017). *MRI-2*, a 69 amino acid peptide, facilitates nonhomologous end joining (NHEJ) DNA repair by interacting with Ku proteins (Slavoff et al. 2014).

### Immune and disease responses

LncRNA-encoded ncPEPs have also emerged as significant diagnostic and prognostic markers in various cancers. *HOX-AS3*, a conserved 53 aa peptide encoded by the putative lncRNA *HOXB-AS3*, prevents tumorigenesis by controlling PKM alternative splicing and metabolic reprogramming in colon cancer cells (Huang 2017). *NoBody*, a 68 amino acid peptide, interacts with proteins involved in mRNA decapping and decay by localizing to mRNA processing bodies and is inversely related to the expression levels of mutant oncogenes (D’Lima et al. 2017).

Several circRNA-encoded peptides have also been found to be involved in cancers, such as glioma, hepatocellular carcinoma, and colorectal cancer (Kong et al. 2020). The circular form of the SNF2 histone linker PHD RING helicase gene, *circ-SHPRH*, is translated into the novel tumor suppressor *SHPRH-146aa*, which has been shown to have an inhibitory effect on tumorigenicity and cell proliferation (Begum et al. 2018, Unk et al. 2006). Encoded by the circular form of the long intergenic noncoding RNA p53-induced transcript (*LINC-PINT*), *PINT-87aa* directly interacts with the polymerase-associated factor complex (PAF1c), which prevents many oncogenes from elongating their transcription (Zhang et al. 2018). Likewise, *FBXW7-185aa*, encoded by *circ-FBXW7*, functions as a cancer suppressor by destabilizing c-Myc, which inhibits cell proliferation and accelerates the cell cycle (Yang et al. 2018). These peptides are often downregulated in glioblastoma, and their corresponding circRNAs are positively correlated with the overall patient survival rate. However, a circRNA-encoded secretory E-cadherin protein variant (*C − E−Cad*) increases EGFR signaling and thus glioblastoma tumorigenicity (Gao et al. 2021).

Primary miRNAs such as *miPEP-200a* and *miPEP-200b*, identified in 2017, control the epithelial‒mesenchymal transition in prostate cancer cells by inhibiting the vimentin-mediated pathway (Ormancey et al. 2023). Immunogenic peptides, such as those from lncRNA β*2.7*, stimulate T-cell responses in individuals with cytomegalovirus infections (Yeasmin et al. 2018). A 44-AA peptide known as an inflammation-modulating micropeptide (IMP) encoded by a putative lncRNA, *lncVLDLR*, interacts with transcriptional coactivators and is dysregulated in patients with type II diabetes and cardiovascular disease (Hartford and Lal 2020).

### Specialized roles in plants

In plant systems, ncPEPs play a role in growth, development, stress responses, and other critical processes. The *Enod40* lncRNA, the earliest eukaryotic micropeptide discovered in plant biology, encodes two short peptides of 12 and 24 AA long, both of which are involved in the organogenesis of root nodules (Röhrig et al. 2002). The *POLARIS* (PLS) gene encodes a 36-AA peptide that influences vascular patterning and cell proliferation (Casson et al. 2002). Additional ncPEPs, such as *ROT4*, *miPEP858a*, and *miPEP408*, have demonstrated potential in enhancing agricultural traits with exogenous application, including the modulation of flowering time and the promotion of root growth, nodulation, and mycorrhizal symbiosis (Wang et al. 2023). Synthetic peptides such as *miPEP171b* and *miPEP165a* have been shown to slow lateral root development and stimulate main root growth, respectively (Lauressergues et al. 2015).

### Computational tools and databases to predict ncRNAs and their encoded peptides

The identification and functional characterization of ncRNAs and their encoded peptides necessitate the use of specialized computational tools because of their small size, noncanonical features and atypical translation mechanisms. The tools listed below in Table 1 employ various strategies, including the prediction of sORFs, assessment of coding potential, promoter recognition, IRES detection and identification of m6A modifications. Notably, tools such as RiboTaper (Calviello et al. 2016), Rp-Bp (Malone et al. 2017), and RibORF (Ji 2018) utilize ribosome profiling data to increase sensitivity in identifying translated regions within ncRNAs.

**Table 1:** Computational tools for the prediction and characterization of noncoding RNAs (ncRNAs) and their encoded peptides (ncPEPs). Each tool is described on the basis of its core strategy, platform type, key features (with predictive performance metrics where reported), input requirements, species applicability, and training dataset

Sl No.	Tools/Platform	Features	Input Requirements	Species Applicability	Training Dataset	References
A. lncRNA Identification Tools						
1	**LGC web server** Web-based	Based on ORF length, GC content; not species specific **| Validation and benchmarking: Validated on large-scale empirical datasets; outperforms existing algorithms with > 90% accuracy across diverse species**	RNA FASTA sequences (> 200 nt)	Multispecies (non-species-specific)	Mixed GENCODE + Ensembl lncRNAs; moderate size	(Wang et al. 2019)
2	**LncDC** Standalone (command-line)	Utilizes an XGBoost model, integrating features extracted from RNA sequences, secondary structures, and translated proteins **| Validation and benchmarking: Validated on human and mouse GENCODE datasets; outperforms earlier lncRNA identification tools**	RNA FASTA + optional secondary-structure annotation	Human, mouse; limited validation in other species	GENCODE human + GENCODE M27 mouse; balanced positive/negative	(Li and Liang 2022)
3	**LncScore** Standalone (command-line)	Utilizes logistic regression to identify sequence and exon features, maximum coding subsequence and ORFs proteins **| Validation and benchmarking: Outperforms CPAT**,** CNCI**,** and PLEK on distinguishing lncRNAs from mRNAs**,** particularly for partial-length transcripts; reports AUC**,** sensitivity**,** and specificity**	RNA FASTA; genome annotation (GTF) optional for exon features	Human, mouse (pretrained models)	GENCODE+ lncRNA transcripts	(Zhao et al. 2016)
**B. sORF Prediction Tools**						
4	**MiPepid** Standalone (command-line)	A machine learning, alignment-free tool; uses Logistic regression with 4-mer features **| Validation and benchmarking: Validated on held-out SmProt sORF test set; alignment-free design improves generalizability over homology-dependent methods**	DNA/RNA FASTA of sORFs (< 100 aa ORFs); requires ATG start codon	Primarily human; applicable to related mammals (mouse, rat)	SmProt database: literature-mined sORFs + Ribo-seq sORFs; negative = miRNA-derived ORFs	(Zhu et al. 2019)
5	**csORF-finder** Standalone (command-line)	Uses trinucleotide deviation from expected mean by integrating the efficient-CapsNet and LightGBM methods **| Validation and benchmarking: Outperforms state-of-the-art sORF prediction methods on multi-species and non-ATG initiation independent test datasets; reports AUC**,** F1**,** and MCC**	RNA/DNA FASTA sequences	Multispecies (human, mouse, zebrafish, Arabidopsis; training covers 7 species)	sORFs.org + multispecies genome annotations; large balanced dataset	(Zhang et al. 2022)
6	**smORFunction** Web-based	Database search followed by identification of the sORFs and pathway enrichment	Protein/peptide FASTA or sORF coordinates	Primarily human (database-dependent)	Curated sORF databases (sORFs.org, SmProt); no independent training	(Ji et al. 2020)
**C. RNA Coding Potential Assessment Tools**						
7	**DeepCPP** Standalone (command-line)	A machine-learning-based alignment tool embedded with a deep neural network; uses nucleotide bias, g-bigap and mDS ranking feature method **| Validation and benchmarking: DeepCPP demonstrates improved performance over CPAT‑ and CPC2‑based approaches through comparative evaluations with CPPred and other state‑of‑the‑art methods**,** using human datasets derived from Ensembl and RefSeq annotations.**	RNA FASTA sequences	Human (primary); limited cross-species validation	GENCODE v27 human; mRNA + lncRNA; random sORF validation	(Zhang et al. 2021)
8	**CPAT** Web-based and Standalone (command-line)	Algorithm-free method using features of ORF size, ORF coverage, Fickett TESTCODE score and hexamer usage bias **| Validation and benchmarking: Validated on human and mouse GENCODE benchmarks; outperforms several contemporary tools on coding potential discrimination**	RNA/DNA FASTA sequences; pretrained models available for human, mouse, zebrafish, fly, worm	Human, mouse, zebrafish, *D. melanogaster*, *C. elegans* (pretrained); custom training for other species	GENCODE + Ensembl; coding + lncRNA per species	(Wang et al. 2013)
9	**CPC2** Web-based	Uses Support Vector Machine (SVM) and features like sequence length, ORF length, and Fickett TESTCODE score **| Validation and benchmarking: Benchmarked across multiple species including human**,** mouse**,** zebrafish**,** fly**,** worm**,** and Arabidopsis; shows competitive performance with CPAT on multi-species datasets**	RNA FASTA sequences (no genome required)	Species-neutral; validated for human, mouse, zebrafish, fly, worm, Arabidopsis	GENCODE + Ensembl + plant databases; per species (smaller than CPAT training set)	(Kang et al. 2017)
10	**RNAsamba** Web-based and Standalone (command-line)	Assessment based on deep neural network; high sensitivity and specificity	RNA FASTA; can handle partial-length ORFs and UTR sequences	Human, mouse, zebrafish, *D. melanogaster*, *S. cerevisiae* (pretrained); retrainable	Union of CPC2 + FEELnc + mRNN human training sets + fragmented ORFs	(Camargo et al. 2020)
11	**PhyloCSF** Standalone (command-line)	Usage of comparative genomics method based on phylogeny; assesses codon substitution patterns across multispecies alignments	Multispecies genome alignments (MAF/PhastCons format); 29-vertebrate or 12-insect alignment	Vertebrates (29-species alignment) and insects (12-species); not suitable for nonconserved/species-specific lncRNAs	12-insect PhastCons; 29-vertebrate genome alignment (UCSC)	(Lin et al. 2011)
12	**ABLNCPP** Standalone (command-line)	Uses mechanism based bidirectional LSTM network integrated with nonoverlapping trinucleotide embedding (NOLTE)	RNA FASTA sequences	Human, mouse; limited cross-species evaluation	GENCODE human + mouse	(Deng et al. 2023)
13	**TransLncPred** Standalone	ML-based model that uses XGBoost to predict translatable lncRNAs	RNA FASTA; optionally Ribo-seq P-site data	Human (primary)	Ribo-seq validated translatable lncRNAs from GENCODE; positive + balanced negative	(Zhang et al. 2023)
14	**CoraL** Standalone (command-line)	Utilizes TextCNN that employs multiple convolutional layers with varying filter sizes to extract features	ncRNA FASTA sequences	Cancer-derived ncRNAs (human cancer cell lines); narrow applicability	Cancer-specific ncRNA databases (TANRIC, lncBook)	(Li et al. 2023)
15	**codLncScape** Standalone (command-line)	Framework for the identification of coding lncRNAs from codLncWeb, codLncDB, and codLncNLP at pancancer data level	lncRNA FASTA + pancancer expression data optional	Human pancancer (TCGA-based)	TCGA pancancer lncRNA expression + curated coding lncRNA database	(Liu et al. 2024)
**D. IRES Site Prediction Tools**						
16	**IRESfinder** Standalone (command-line)	Based on a logit model of 19 framed k-mer features; includes plotting via R software **| Validation and benchmarking: Validated on human cellular IRESs from IRESbase and IRESite; limited by small training dataset**	RNA/DNA sequences (5’ UTR recommended; <174 nt window)	Human IRES only (training data from IRESbase — human cellular IRESs)	Human IRESs from IRESbase + IRESite; small dataset	(Zhao et al. 2018)
17	**IRESpy** Web-based	Based on sequence features such as k-mer words, structural features such as QMFE incorporated with XGBoost model; useful for genome-wide comparisons **| Validation and benchmarking: Validated on high-throughput IRES screen data; suitable for genome-wide comparisons**	RNA sequences (≤ 174 nt windows; sliding window for longer sequences)	Viral and cellular IRES; validated on human + selected viral genomes	High-throughput IRES screen data; thousands of experimentally validated IRES	(Wang and Gribskov 2019)
18	**DeepIRES** Standalone (command-line)	A hybrid deep learning-based model that dilates 1D convolutional neural network blocks, a self-attention module, and bidirectional gated recurrent units **| Validation and benchmarking: Hybrid deep learning architecture; outperforms IRESfinder and IRESpy on balanced cellular and viral IRES test set**	RNA sequences; fixed 174 nt window (padding/truncation applied)	Cellular and viral IRES (human and viral genomes); best validated for human cellular + viral sequences	Experimentally validated IRES + IRESbase; balanced positive/negative	(Zhao et al. 2024)
**E. Ribosome Profiling/ORF Detection Tools**						
19	**RiboTaper** Standalone (command-line)	Uses Thomson’s multitaper F test to detect 3-nt periodicity of ribosome footprints; includes de novo ORF finding	Ribo-seq BAM files + RNA-seq BAM (required); genome annotation (GTF); requires ≥ 50% RPF-supported P-sites	Eukaryotes (human, mouse, yeast benchmark); NOT compatible with bacterial annotations	Validated on human HEK293 and yeast Ribo-seq datasets	(Calviello et al. 2016)
20	**Rp-Bp** Standalone (command-line)	Uses unsupervised Bayesian probability modeling to infer ribosome P-sites and translated regions; can detect novel and short ORFs effectively	Ribo-seq BAM + RNA-seq BAM; genome annotation; requires custom preprocessing	Eukaryotes (human, mouse, yeast tested); Not compatible with bacterial annotations	Human + yeast Ribo-seq datasets; Bayes factor ranking for ORF confidence	(Malone et al. 2017)
21	**RibORF** Standalone (command-line)	Feature-driven SVM classifier based on read distribution features representing active translation, including 3-nt periodicity and coverage uniformity	Ribo-seq BAM files + transcript annotation; model training on known ORFs (supervised)	Eukaryotes (human, mouse validated); Not compatible with bacterial data	Annotated human/mouse CDS ORFs from GENCODE used as training positives	(Ji and RibORF 2018)
**F. m6A Site Prediction Tools**						
22	**DNN-m6A** Web-based and Standalone (command-line)	A deep learning-based tool that utilizes a deep neural network framework and works across multiple species; trained via a 5-fold cross-validation technique **| Validation and benchmarking: Cross-species validated across human**,** mouse**,** zebrafish and others using 5-fold cross-validation**	RNA sequences around candidate adenosine sites (101 bp window recommended)	Multispecies: human, mouse, zebrafish + others (cross-species validated)	miCLIP-seq + MeRIP-seq datasets across multiple species; species-balanced training	(Zhang et al. 2021)
23	**DeepM6ASeq-EL** Standalone (command-line)	Utilizes an ensemble of LSTM and CNN classifiers; limited to human m6A sites **| Validation and benchmarking: Outperforms single-classifier baselines on human m6A site prediction**	RNA FASTA sequences (101 bp window centered on candidate A site)	Human only (trained and validated exclusively on human miCLIP-seq data)	Human miCLIP-seq; balanced positive/negative	(Chen et al. 2022)
**G. circRNA Identification Tools**						
24	**CIRIlong** Standalone (command-line)	Python-based tool for the identification of circRNAs in long-read sequencing data; uses k-mer-based repetitive identification, consensus sequencing calculation and BSJ determination | **Validation and benchmarking**: **Benchmarked against simulated and real datasets; achieves substantially higher circRNA enrichment compared to Illumina-based strategies**	Long-read sequencing data (Oxford Nanopore/PacBio); genome FASTA + GTF annotation	Applicable to any species with genome reference; validated in human and mouse	Human and mouse long-read transcriptome datasets; benchmarked against simulated + real data	(Hou et al. 2023)
25	**Isocirc** Standalone (command-line)	Computational pipeline to characterize full-length circRNA isoforms from long-read data	Long-read sequencing data (Nanopore/PacBio); genome annotation (GTF)	Human (validated); adaptable to other species with genome reference	Human long-read RNA-seq from brain/heart tissues; benchmarked against simulated circRNA reads	(Xin et al. 2021)
26	**CIRI2** Standalone (command-line)	Employs an adapted maximum likelihood estimation (MLE) based on multiple seed matching to identify back-spliced junction (BSJ) reads and filter false positives; supports both single-end and paired-end modes; optional GTF for annotation-dependent detection **| Validation and benchmarking: Highest F**_**1**_ **score among all competing tools on Hs68 and HEK293 datasets; outperforms all algorithms across all RNase R enrichment thresholds**	SAM alignment file generated by BWA-MEM (required); FASTA reference genome (required); GTF annotation file (optional)	Validated in human and mouse; adaptable to other species with a reference genome	Human Hs68 cell line RNA-seq with and without RNase R treatment; benchmarked against 9 other circRNA detection tools using F1 score, sensitivity, and FDR	(Gao et al. 2018)
27	**UROBORUS** Standalone (command-line)	Perl-based pipeline using TopHat and Bowtie; employs a 20 bp artificial paired-end seed extraction strategy from unmapped reads, handling both balanced mapped junction (BMJ) and unbalanced mapped junction (UMJ) reads; capable of detecting low-expression circRNAs without RNase R treatment **| Validation and benchmarking: Validated on 24 of 27 randomly selected circRNA candidates by RT-PCR and Sanger sequencing in glioma tissue**	Total RNA-seq (rRNA-depleted, PolyA-minus); paired-end reads; human reference genome (hg19)	Human (validated in glioma and normal brain tissue); adaptable to other species with a reference genome	Human glioblastoma (GBM, *n* = 20), oligodendroglioma (*n* = 7), and normal brain tissue (*n* = 19); Illumina HiSeq 2000, 50-bp PE reads; 55–91.5 million reads per sample	(Song et al. 2016)

*BSJ* back-spliced junction, *BMJ* balanced mapped junction, *UMJ* unbalanced mapped junction, *FDR* false discovery rate, *GTF* gene transfer format, *MLE* maximum likelihood estimation, *SVM* support vector machine, *LSTM* long short-term memory, *CNN* convolutional neural network, *NOLTE* nonoverlapping trinucleotide embedding, *QMFE* quasiminimum free energy

**Table 2:** Summary of publicly available databases for ncRNA-encoded peptides (ncPEPs). Each entry provides a description of the database content, species covered, the access URL, download and programmatic accessibility, and an indication of data volume and curation status to facilitate resource selection for computational and machine learning applications

Database	Content description	Species covered	URL	Availability & Download	Data Volume & Curation
ncEP (Liu et al. 2020)	Experimentally validated ncRNA-encoded proteins/peptides from published articles	18 species incl. *H. sapiens*, *A. thaliana*, *D. melanogaster*	http://www.jianglab.cn/ncEP/	Freely accessible; supports search, browse, download, and user submission; no API reported	80 entries covering 74 proteins/peptides manually curated; low-throughput experimentally validated)
SPENCER (Luo et al. 2022)	ncPEPs across 15 cancer types identified by MS-based proteomics	*H. sapiens*	http://spencer.renlab.org	Freely accessible; browse and download available; no API reported	more than 29,000 ncRNA-encoded peptides; large volume, computationally identified + cross-validated
FuncPEP (Mohapatra et al. 2024)	Functionally validated ncRNA-encoded peptides from published articles	14 species incl. *H. sapiens*, *A. thaliana*, *D. melanogaster*, *E. coli*	https://bioinformatics.mdanderson.org/Supplements/FuncPEP/	Freely accessible; browse and download; no API reported	152 functional ncPEPs, of which 40 are novel entries, highly curated (experimentally validated and functionally characterized)
TransLnc (Lv et al. 2022)	Translatable lncRNA peptides with tumor neoantigen predictions	*H. sapiens*, *M. musculus*, *R. norvegicus*	https://bio-bigdata.hrbmu.edu.cn/TransLnc/	Freely accessible; download available	Integrates 6 evidence types; manually curated + computational prediction; moderate volume
LncPEP (Liu et al. 2022)	Predicted peptides from lncRNAs via coding potential evaluation	39 species across plant and animal taxa	http://www.shenglilabs.com/LncPep/	Freely accessible; full dataset download available; BLAST and prediction tools	10,580,228 peptides translated from 883,804 lncRNAs across 39 species.
smProt (Li et al. 2021)	Small proteins (< 100 aa) from Ribo-seq, MS, and literature	8 species, 291 cell lines/tissues	http://bigdata.ibp.ac.cn/SmProt/	Freely accessible; download page available; BLAST service; no API reported	638,958 unique small proteins curated from 3,165,229 primary records
NCPbook (Sami et al. 2024)	Noncanonical peptides from MS, Ribo-seq, and molecular experiments	29 species (14 plants, 7 animals, 8 microbes)	https://ncp.wiki/ncpbook/	Freely accessible; download links available by species; BLAST and JBrowse	180,676 NCPs including 9,166 functionally characterized; large volume, multi-evidence

### Databases for noncoding RNA-encoded peptides

Biological databases are vital sources of biological data, encompassing everything from complicated systems such as metabolic pathways to genetic sequences and protein structures. Large volumes of data from experiments and literature are organized in these databases, providing scientists with accessible platforms worldwide. Table 2 summarizes key databases currently available for ncPEP research.

1. **ncEP**.

The ncEP database records information on peptides translated by ncRNAs, including lncRNAs, circRNAs, and pri-miRNAs, which have been curated manually from research articles (Liu et al. 2020). The entries cover peptides across several species along with their function and experimental evidence. The database is cross-referenced with the EntrezID for the ncRNA along with the PMID for the publication. Each peptide record is presented with the sequence of the peptide, length, information of the ncRNA encoding the peptide, and subcellular localization along with the cell lines of expression.

2. **SPENCER**.

SPENCER provides a platform for exploring functional ncPEPs in cancer derived from mass spectrometry data analyzed via MaxQuant (Luo et al. 2022). The peptides are categorized across 15 different cancer types and grouped according to their expression in the cancer environment, including tumor specific, upregulated, and downregulated groups. The database provides information on ncPEPs from five different RNA types but is limited to human data only.

3. **FuncPEP**.

FuncPEP focuses on ncPEPs with validated functional roles in cellular processes or disease contexts (Mohapatra et al. 2024). Data are from studies involving mass spectrometry, western blotting, ribosome profiling and functional assays. Entries include the name of the ncPEP, function, length, sequence, molecular weight, associated ncRNA and external links to the NCBI and Pfam databases.

4. **TransLnc**.

TransLnc is a database specifically focused on translatable lncRNA peptides in humans, rats and mice (Lv et al. 2022). It integrates six types of direct and indirect experimental evidence, namely, manual curation, ribosome occupancy, lncORF, IRES, m6A, and mass spectrometric evidence. The database also includes tumor neoantigen predictions, offering insights into immunotherapeutic potential and tissue-specific expression profiles.

5. **LncPEP**.

LncPEP provides comprehensive data on lncRNA-encoded peptides across 39 species (Liu et al. 2022). It combines five different lines of evidence, including CPC2, CPAT, Ribo-seq, m6A and translation initiation site prediction. The database also includes peptides derived from experimental validation found in published articles. The platform supports the BLAST search option and peptide prediction from FASTA sequences. The entries in the database provide brief information on the peptide, length of the peptide and sequence, along with the host RNA and evidential information.

6. **smProt**.

smProt catalogs small proteins (< 100 amino acids) derived from untranslated regions and noncoding regions (Li et al. 2021). Data are compiled from Ribo-seq predictions, literature curation, existing databases, and mass spectrometry evidence across eight species and 291 cell lines. Annotations include genomic location, function, disease associations, and variant information with BLAST search functionality.

7. **NCPbook**.

The NCPbook includes a vast collection of noncanonical peptides, including ncPEPs, from 29 species using data from publicly available databases and literature. The entries are supported by evidence from mass spectrometry, molecular experiments or ribosome profiling (Sami et al. 2024).

In addition to databases that directly catalog ncPEPs, complementary circRNA annotation resources provide important upstream support for circRNA-encoded peptide discovery. circRNADb is a comprehensive repository of 32,914 nonredundant human exonic circRNAs annotated with genomic coordinates, exon splicing information, predicted open reading frames (ORFs), and internal ribosome entry site (IRES) element molecular features that are prerequisites for peptide translation from circRNAs (Chen et al. 2016). A subset of entries is further supported by mass spectrometry-based protein expression evidence, with 46 circRNAs from 37 genes confirmed to encode proteins in human brain tissue. While circRNADb does not catalog peptide sequences directly in the manner of ncPEP-specific databases, its ORF and IRES annotations make it an essential starting point for researchers seeking to identify candidate circRNA-encoded peptides prior to experimental validation.

### Evolving strategies for the computational and experimental identification of noncoding RNA-encoded peptides

The identification of ncPEPs has not emerged from a single methodology but from the progressive convergence of three distinct investigative tiers. Early efforts relied exclusively on sequence-based computational prediction leveraging intrinsic transcript features, homology and evolutionary conservation to distinguish coding from noncoding transcripts. While these approaches established a critical foundation, their inherent limitation in recognizing sORFs within noncoding transcripts necessitated a methodological shift toward translation-level evidence, realized through ribosome profiling and allied sequencing strategies. The limitations of translation evidence alone, in turn, drove the adoption of proteogenomics and the integration of genomic, transcriptomic, and MS-based proteomics to provide definitive protein-level confirmation. The following subsections trace this methodological progression, highlighting the specific limitations that drove each transition and the complementary strengths that warrant their combined application in contemporary ncPEP discovery pipelines

### Tier 1 - sequence-level evidence

High-throughput RNA sequencing (RNA-seq) has revolutionized transcriptomic research by enabling the detection of both annotated and novel transcripts, including those with regulatory and coding potential. Most RNA-seq studies employ short-read sequencing, with transcript assembly conducted via either reference-guided or de novo methods (Stark et al. 2019, Raghavan et al. 2022). De novo assembly offers an advantage in capturing full-length transcripts and identifying variations arising from alternative splicing and posttranscriptional modifications (Hari et al. 2022). Since mRNA levels frequently have a poor correlation with protein abundance due to substantial translational and posttranslational regulation, RNA-seq measures transcript abundance but does not directly demonstrate translation

With a rapid increase in the volume of transcriptomic data, researchers have applied coding potential estimation, which was originally developed to detect novel coding genes, to distinguish coding RNAs from their noncoding counterparts (Choi et al. 2019). These methods rely on intrinsic sequence characteristics and evolutionary features, such as ORF length, substitution ratio, sequence conservation, homology to known protein sequences, secondary structure and nucleotide composition. Intrinsic sequence-based predictors, including CPAT (logistic regression), CNCI, PLEK, and PORTRAIT, rely primarily on ORF statistics and compositional features without cross-species alignment. However, several studies have reported that such tools show reduced sensitivity toward sORFs due to weak coding signatures and training biases toward canonical protein-coding genes (Bazzini et al. 2014, Ruiz-Orera et al. 2014). Homology-dependent approaches, such as CPC and CONC, incorporate BLAST-based similarity searches alongside machine learning classifiers. While effective for conserved proteins, homology-based frameworks inherently fail to detect recently evolved, species specific, or rapidly diverging peptides that lack detectable database matches (Ruiz-Orera et al. 2020). Conservation-based frameworks, including PhyloCSF, assess codon substitution patterns across multispecies alignments to identify evolutionary constraints at the coding level. Although powerful for conserved genes, these methods may overlook young or poorly conserved sORFs and thus limit their applicability in less-characterized genomes (Bazzini et al. 2014). Finally, integrative machine learning models, such as iSeeRNA, and ensemble-based methods, such as lncRNA-ID, COME and lncRNA-MFDL, combine multiple sequence and evolutionary features to improve discrimination accuracy. However, ensemble classifiers remain dependent on the quality and balance of training datasets and may suffer from overfitting or reduced generalizability when applied to newly annotated transcriptomes (Han et al. 2016)

A study by Cao et al. investigated the function of lncRNA-encoded transmembrane peptides in glioma using RNA-seq data from The Cancer Genome Atlas (TCGA) through differential expression analysis and coding potential assessment via CPC and CPC2. Structural modeling and molecular dynamics with free energy profiling revealed that *DLEU1*-derived ORF1 formed a functional water channel, highlighting the critical roles played by lncRNA-encoded peptides in glioma cell physiology (Cao et al. 2021) (Fig. 4a)

### Tier 2 - translational evidence

**Fig. 3:**
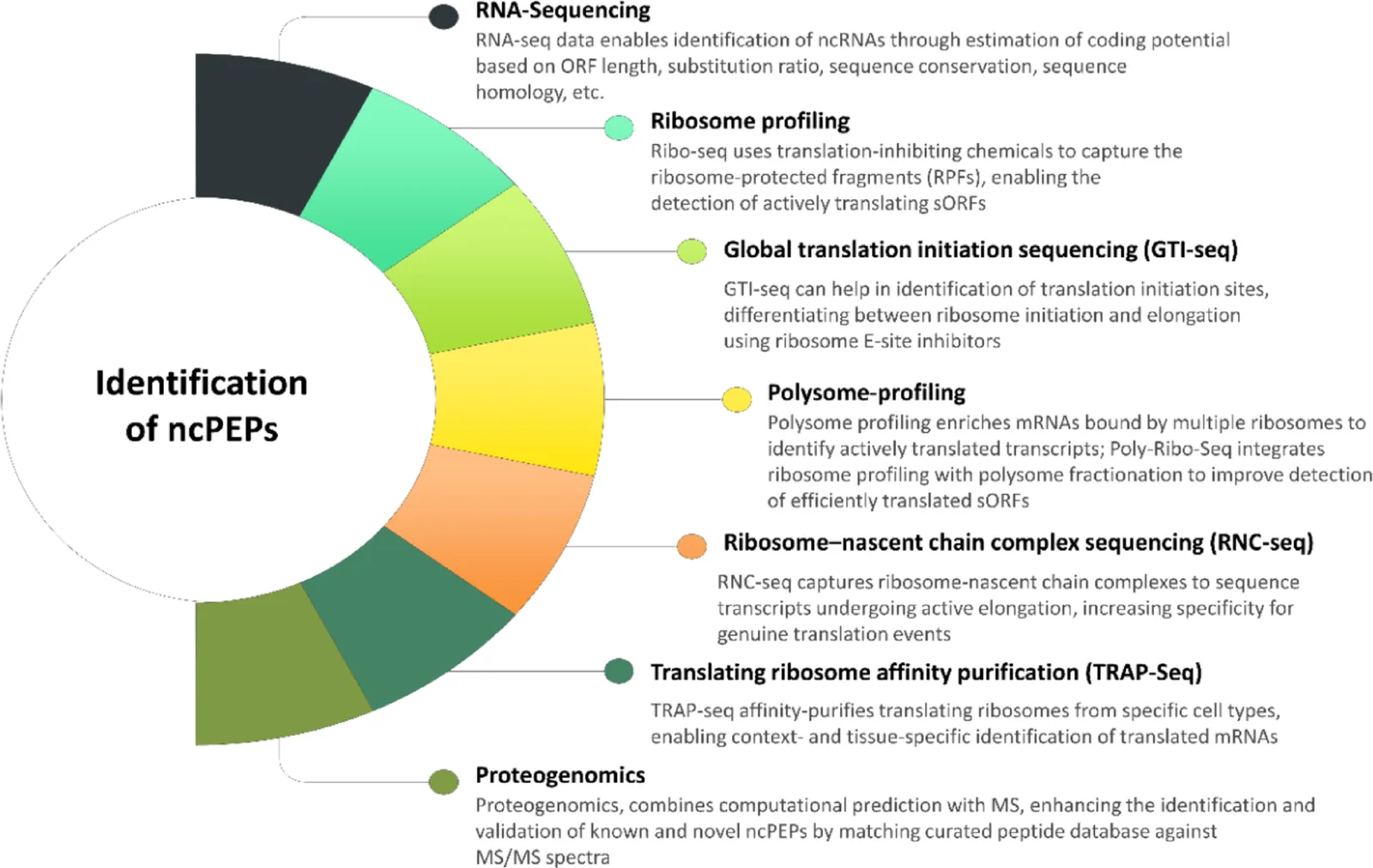
Summary of the multi-omics approaches used for the identification of ncRNA-encoded peptides (ncPEPs). The figure illustrates the Tier 2 translational evidence strategies and the Tier 3 protein-level evidence strategy of proteogenomics. Each approach provides a complementary layer of evidence for transcript expression, ribosome engagement, and peptide detection, collectively enabling the progressive validation of computationally predicted ncPEPs

 While these traditional computational tools have successfully distinguished coding from noncoding transcripts, they often overlook sORFs as a feature of noncoding transcripts, which may encode functional ncPEPs. Complementary translation-omics methods, such as ribosome profiling (Ribo-Seq), polysome profiling, ribosome–nascent chain complex sequencing (RNC-Seq) and translating ribosome affinity purification (TRAP-Seq), enhance the detection of actively translated sORFs (Pan et al. 2022) (Fig. 3)

 Ribosome profiling (Ribo-Seq), introduced by Ingolia et al. in 2009, captures ribosome-protected RNA fragments (RPFs) to provide a snapshot of global translation (Ingolia et al. 2011). While early metrics such as translation efficiency (TE) were limited in distinguishing active translation from nonspecific ribosome binding, the discovery of three-nucleotide periodicity, a hallmark of active translation, marked a significant advancement (Choi et al. 2019, Bazzini et al. 2014, Ingolia et al. 2011). Tools such as RiboTaper, Rp-Bp, and RibORF leverage this periodicity to enhance the detection of translated sORFs (Calviello et al. 2016, Malone et al. 2017, Ji 2018). Additionally, a specialized variant of Ribo-Seq used in combination is global translation initiation sequencing (GTI-seq). It distinguishes between ribosome initiation and elongation via two translation inhibitors, cycloheximide (CHX) and lactimidomycin (LTM), which readily bind to translating ribosomes and free E-sites, respectively (Choi et al. 2019). Nonetheless, factors such as nuclease digestion biases and stalled ribosomes can cause misleading signals, leading to difficulty in differentiating true translation from background noise

Polysome profiling, a rapid and more cost-effective screening method to assess the global translation status, serves as a first-pass screen prior to Ribo-seq. This technique separates mRNAs based on the number of engaged ribosomes using sucrose gradient ultracentrifugation (Pan et al. 2022). Nevertheless, this technique lacks codon-level resolution and is susceptible to variability introduced by sucrose gradient fractionation. In addition, polysome profiling can be used in combination with Ribo-seq, also known as poly-Ribo-Seq. It involves performing ribosome footprinting specifically on the polysome fractions and enhances sORF detection by focusing on the multiribosome complexes (Aspden et al. 2014). On the other hand, RNC-seq is a full-length, sequencing-based extension of polysome profiling, providing a direct quantification of abundance and type of translating RNAs. It captures actively translating full-length RNAs, enabling isoform and splice-variant resolution, but shows lower precision than Ribo-seq (Pan et al. 2022).

 A cell type-specific approach to profile translating mRNAs is TRAP-seq, wherein enhanced green fluorescent protein (EGFP)-tagged ribosomes and associated mRNAs are affinity purified from tissue lysates using anti-EGFP antibodies (Heiman et al. 2014). It shows high sensitivity for low-abundance RNAs in defined populations and has been used to map actively translated transcripts in cardiomyocytes (Yan et al. 2021). However, it cannot identify specific sORFs and requires transgenic or viral delivery of tagged proteins, which limits its applicability across experimental systems

### Tier 3 - protein-level evidence

**Fig. 4:**
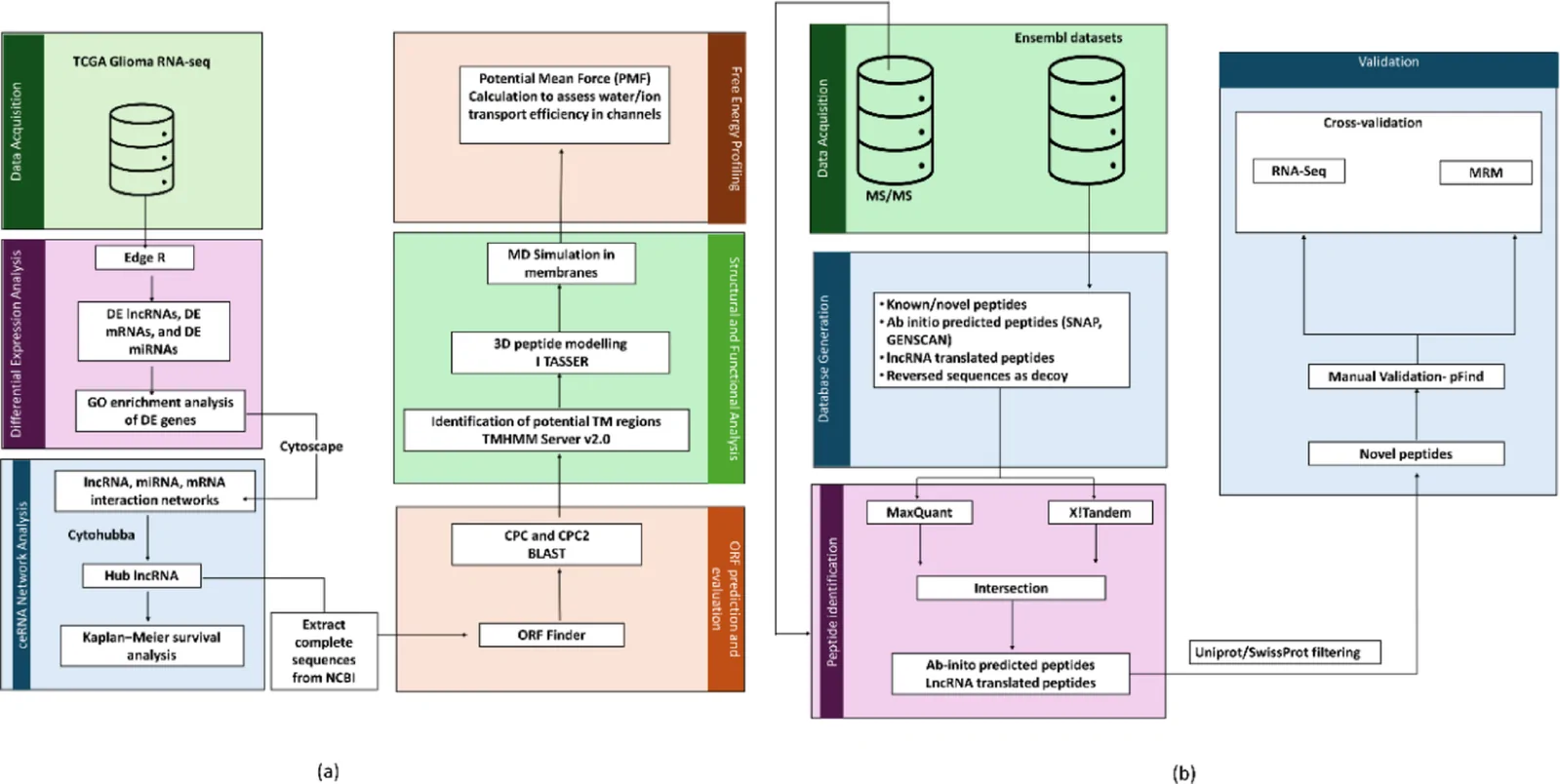
Overview of the established workflows for the identification and validation of ncPEPs. **a** Differential expression analysis of glioma RNA-seq data from TCGA was performed using EdgeR, followed by assessment of coding potential using CPC and CPC2. Structural-functional analysis using I-TASSER and PMF calculations provides insights into transmembrane channel efficiency and functional characterization. **b** MS/MS (tandem mass spectrometry) data and RNA-seq data are integrated to identify ncPEPs with tools such as MaxQuant and X! Tandem followed by cross-validation using RNA-seq and MRM

A specialized subfield known as proteogenomics integrates genomics or transcriptomic data with MS-based proteomics to provide direct evidence of peptide translation. It involves creating a custom peptide database from predicted sORFs in cDNA sequences and RNA-seq data, which are then matched against the MS/MS spectra to provide direct protein-level evidence of translation, while the unmatched peptides are identified as novel (Nesvizhskii 2014, Sun et al. 2014). For example, a study by Sun et al. identified six peptides encoded by ab initio-predicted genes that had not been validated previously. The approach involved matching a database curated from Ensembl datasets against MS/MS spectra from HeLa cells using MaxQuant and X! Tandem, with validation via RNA-seq and multiple reaction monitoring (MRM) (Sun et al. 2014) (Fig. 4**b**). This underscores the utility of proteogenomic approaches in the refinement of human gene annotation

Recent developments in the fields of MS and RNA-Seq have led to the use of proteogenomics to detect novel peptides, splice variants, and fusion products that serve as biomarkers for the diagnosis and prognosis of tumors (Hari et al. 2022). However, challenges remain, as the standard MS protocols are biased toward more abundant larger peptides, and many sORFs could be undetected due to their rapid degradation (Makarewich and Olson 2017). Another major limitation arises from the database-dependent nature of proteogenomics, including the lack of many sample‑specific variants and novel coding regions in the reference protein databases, reducing their chance of detection (Nesvizhskii 2014). Moreover, expanding the database size increases the discovery potential but inflates the false discovery rate, complicating confident identification of true peptides (Woo et al. 2015). Nevertheless, sample-specific databases derived from RNA-seq data offer promising solutions for capturing transcriptional and posttranscriptional variability (Hari et al. 2022). Numerous tools and pipelines have been designed to support proteogenomic workflows, including PGA (Wen et al. 2016), JUMPg (Li et al. 2016), IPAW (Zhu et al. 2018) and ProteomeGenerator (Cifani et al. 2018). Platforms such as FragPipe (https://fragpipe.nesvilab.org/) provide tools for comprehensive solutions for MS-based proteomic analysis, facilitating the discovery of ncPEPs in cancer and other biological contexts

## Discussion

Recent discoveries revealing the extraordinary ability of sORFs within ncRNAs to encode ncPEPs have challenged the traditional view of coding versus noncoding transcripts. Despite their tiny size, ncPEPs have been implicated in a wide range of biological processes, including cellular development, cellular differentiation, energy metabolism, and homeostasis (Pauli et al. 2014, Stein et al. 2018)

onventional ncPEP prediction tools, which rely on estimation of coding potential through features such as ORF length, sequence conservation, and homology, are increasingly being complemented by advanced strategies that consider IRESs, m6A modifications, and non-AUG initiation codons. Many predicted sORFs are evolutionarily young and, as a result, may be missed by conservation-based tools. Furthermore, biases such as ORF length and AUG-start codons may cause sORFs to be overlooked by tools that rely solely on intrinsic exon characteristics. To address these limitations, newer tools are increasingly integrating experimental data such as Ribo-Seq, GTI-seq, and MS data, which provide evidence at the translational level. In recent years, ab initio training, machine learning, and homology-based searches have incorporated experimentally validated sORFs to increase the accuracy and robustness of sORF prediction methods (Zhu et al. 2019, Cheng et al. 2011). The next phase of ncPEP research will likely be driven by multi‑modal machine learning models that integrate sequence features with translatome and proteogenomic evidence. Tools that combine multiple Ribo‑seq–derived scores, such as recent ribosome‑profiling-assisted proteogenomics frameworks (for e.g., Rp3), already improve sensitivity and confidence for microprotein discovery compared with single‑signal callers alone (Vieira de Souza et al. 2024). Deep learning architectures have become state‑of‑the‑art for distinguishing coding from noncoding transcripts, convolutional and recurrent models such as LncRNAnet, and newer transformer‑based approaches, consistently outperforming traditional coding‑potential indices and are beginning to recover misannotated lncRNAs with short ORFs (Baek et al. 2018)

As the field continues to evolve, the development of comprehensive and well-curated databases becomes essential. Resources such as NCPbook, smPROT, LncPEP and TransLnc offer both computational and experimental evidence for ncPEPs, whereas databases such as Spencer provide cancer-specific classifications, reflecting the growing recognition of ncPEPs in tumorigenesis. The future lies in the use of large models, such as Huawei’s domain-specific PanGu Drug Model, which may be tailored to enable the rapid identification of ncPEPs from vast transcriptomic datasets with predictive functional assignment for streamlining database organization (Lin et al. 2023). Furthermore, AI-driven platforms such as GeneWhisperer (Li et al. 2025) and DeepGOPlus (Xu and Cui 2025) may accelerate functional annotation and database curation by integrating sequence homology, motif detection, and literature mining. Human-centered AI frameworks such as HAICoGA have been proposed to overcome model biases that involve human validation loops and AI-assisted workflows to leverage the strengths of both humans and AI for accurate genome annotation (Li et al. 2025)

 However, these machine‑learning approaches face important limitations. Labeled datasets specific to ncPEPs are still small and noisy, and only a minority of predicted sORFs have been biochemically validated. Many lncRNAs appear to exert both RNA and peptide‑mediated functions, and there are few curated negative sets, all of which constrain supervised training for coding‑potential and sORF‑detection models. In addition, the Ribo‑seq and MS data used as training targets contain systematic artifacts such as scanning ribosomes, digestion and coverage biases, and under‑detection of sORFs that can propagate into model predictions if not explicitly modeled (Kong et al. 2025, Limbu et al. 2024). Deep‑learning frameworks for lncRNAs also show species- and tissue‑specific overfitting, sensitivity to confounders such as ORF length and GC content, and limited interpretability, raising questions about how well models trained in one context will generalize to others relevant for ncPEPs (Ammunét et al. 2022)

While the de novo design of ncPEPs is unexplored, computational platforms such as RFdiffusion and Rosetta generate ncPEP-sized miniproteins (20–100 aa helical bundles) that bind diverse targets (GPCRs, proteases) with nM affinity, providing a blueprint for ncPEP-inspired mimics (Muratspahić et al. 2025, Watson et al. 2023). These advances position ncPEPs as templates for next-generation mini-protein therapeutics. Despite these advancements, ncPEP research remains in its early stages. Challenges persist in the detection, validation and functional characterization of these small peptides. Since ncRNAs can function both as regulatory RNA molecules and translated functional peptides (Hubé and Francastel 2018), this additional level of complexity expands the scope of their potential in human disorders

## Conclusion

The discovery and functional characterization of ncPEPs represents a rapidly advancing frontier in molecular biology. In silico strategies, supported by predictive algorithms and curated databases, have significantly contributed to uncovering the hidden proteomic landscape encoded within ncRNAs. As our understanding of ncPEP biology deepens, their involvement in critical cellular pathways increasingly highlights their clinical utility. One of the emerging areas of interest is the role of ncPEPs as tumor biomarkers and therapeutic targets that could facilitate the early diagnosis and treatment of human illnesses (Zhang et al. 2023). Translating these discoveries into clinical applications will require the integration of AI-driven annotation frameworks with expert human curation, ensuring accurate interpretation of the hidden proteomic landscape encoded within ncRNAs

## Data Availability

No datasets were generated or analysed during the current study.

## Abbreviations

AA: Amino Acid
ABLNCPP: Association of Binding Locations in Noncoding Potential Prediction
ATPase: Adenosine Triphosphatase
CapsNet: Capsule Networks
cDNA: Complementary DNA
C − E−Cad: circRNA-encoded E-cadherin
CHX: Cycloheximide
CI: Coding Index
circRNA: Circular RNA
circ-SHPRH: Circular SHPRH RNA
c-Myc: Cellular Myc
CNCI: Coding-Noncoding Index
CNN: Convolutional Neural Networks
CPAT: Coding Potential Assessment Tool
CPC: Coding Potential Calculator
DeepCPP: Deep neural network for coding potential prediction
DLEU1: Deleted in Lymphocytic Leukemia 1 (lncRNA)
DNA: Deoxyribonucleic Acid
DWORF: Dwarf Open Reading Frame
EGFR: Epidermal Growth Factor Receptor
EMT: Epithelial–mesenchymal transition
FASTA: FAST-All
FBXW7: F-box/WD Repeat-Containing Protein 7
FuncPEP: Functional Non-Coding RNA Encoded Peptides
GC: Guanine-Cytosine Content
GO: Gene Ontology
GTI-seq: Global translation initiation sequencing
HLA: Human Leukocyte Antigen
HOXB: Homeobox B (gene family)
IMP: Inflammation-Modulating Micropeptide
IPAW: Integrated Proteogenomic Analysis Workflow
IRES: Internal Ribosome Entry Site
LightGBM: Light Gradient Boosting Machine
LINC-PINT: Long Intergenic Non-Protein Coding RNA P53 Induced Transcript
LncDC: Long noncoding RNA detection
lncRNA: Long Non-Coding RNA
LSTM: Long Short-Term Memory (a type of neural network)
LTM: Lactimidomycin
m6A: N6-Methyladenosine
mDS: Model Distance Score
miPEP: MicroRNA Encoded Peptide
MiPepid: Micropeptide Identification Tool
miRNA: MicroRNA
MLN: Myoregulin
MRM: Multiple Reaction Monitoring
MS: Mass Spectrometry
MS/MS: Tandem Mass Spectrometry
Mtln: Mitoregulin
mTORC1: Mammalian Target of Rapamycin Complex 1
ncEP: Non-Coding RNA Encoded Peptides or proteins
ncPEPs: Non-Coding RNA Encoded Peptides
NCPs: Noncanonical peptides
ncRNA: Non-Coding RNA
ORF: Open reading Frame
PAF1c: Polymerase-Associated Factor 1 Complex
Pfam: Protein families database
PGA: Proteogenomics Analysis
PHD: Plant Homeodomain
PhyloCSF: Phylogenetic Codon Substitution Frequency
piRNA: Piwi-Interacting RNA
PKM: Pyruvate Kinase
PLS: POLARIS gene
PMF: Potential mean force
Poly(A) Tail: Polyadenylate Tail
RNA: Ribonucleic Acid
RNA-seq: RNA Sequencing
Rp-bp: Ribosome profiling with Bayesian predictions
RPF: Ribosome Protected Fragment
rRNA: Ribosomal RNA
SERCA: Sarco/Endoplasmic Reticulum Calcium ATPase
SHPRH: SNF2 Histone Linker PHD RING Helicase
smPROT: Small Proteins database
SNF: Switch/Sucrose Non-Fermentable
snRNA: Small Nuclear RNA
sORF: Short Open Reading Frame
SPAR: Small Regulatory Polypeptide of Amino Acid Response
SVM: Support Vector Machine
TCGA: The Cancer Genome Atlas Program
textCNN: Text Convolutional Neural Network
tRNA: Transfer RNA
XGBoost: Extreme Gradient Boosting
